# Why Was the 2009 Influenza Pandemic in England So Small?

**DOI:** 10.1371/journal.pone.0030223

**Published:** 2012-02-10

**Authors:** Ruben J. Kubiak, Angela R. McLean

**Affiliations:** Institute for Emerging Infections, Oxford Martin School, Department of Zoology, University of Oxford, Oxford, United Kingdom; Northeastern University, United States of America

## Abstract

The “Swine flu” pandemic of 2009 caused world-wide infections and deaths. Early efforts to understand its rate of spread were used to predict the probable future number of cases, but by the end of 2009 it was clear that these predictions had substantially overestimated the pandemic's eventual impact. In England, the Health Protection Agency made announcements of the number of cases of disease, which turned out to be surprisingly low for an influenza pandemic. The agency also carried out a serological survey half-way through the English epidemic. In this study, we use a mathematical model to reconcile early estimates of the rate of spread of infection, weekly data on the number of cases in the 2009 epidemic in England and the serological status of the English population at the end of the first pandemic wave. Our results reveal that if there are around 19 infections (i.e., seroconverters) for every reported case then the three data-sets are entirely consistent with each other. We go on to discuss when in the epidemic such a high ratio of seroconverters to cases of disease might have been detected, either through patterns in the case reports or through even earlier serological surveys.

## Introduction

In the spring of 2009 an influenza pandemic arose in North America. The first ‘Swine flu’ influenza A(H1N1) cases were recorded in March 2009 in Mexico [1]. Infection soon became a global phenomenon: the first laboratory confirmed cases in the UK were on 27 April [2], and by the end of May more than 50 countries had confirmed cases. The World Health Organisation formally declared the outbreak a pandemic on 11 June 2009 [3].

As the pandemic spread around the world it was natural to ask “how bad will it be?”, and attempts to forecast epidemic impacts were rapidly assembled, broadcast and then published. Several of these studies focused on estimating the new influenza's basic reproductive number 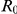, defined as the number of secondary cases caused by each case as an epidemic spreads into a population with no pre-existing immunity [4]. A related parameter, the effective reproductive number, 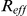, pertains when an epidemic spreads in a population in which some proportion are already immune [5].

Estimates of the basic reproductive number ranged from 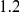 to 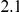 (see Table 1). A mid-range estimate of 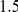 implied that the epidemic would grow until 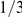 of the population were infected or immune, then turnover and come to an end when around 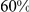 of the population were immune. If all infections lead to illness prior to the acquisition of immunity this implied that a very large fraction of the population would become ill during the pandemic's early waves. Some alarming predictions were indeed made, particularly for the UK.

**Table 1:** Reproductive numbers estimates for influenza A(H1N1) from several independent studies.

Reproductive number estimates
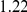 – 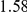 (CI: 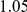 – 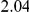 ) [1]
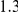 – 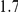 [17]
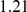 – 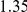 [18]
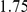 (CI: 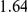 – 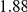 ) [19]
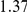 (CI: 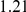 – 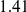 ) 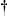 [20]
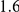 (CI: 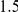 – 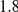 ) 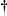 [21]
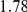 – 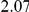 (CI: 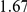 – 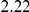 ) [22]
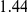 (CI: 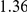 – 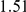 ) 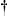 [23]
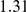 (CI: 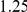 – 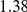 ) [24]

A range of possible reproductive numbers is presented when multiple estimations were made. Estimates marked with 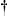 are the effective reproductive number 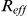, while unmarked ones are the basic reproductive number 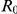. The confidence interval (CI) is given wherever possible.

In England, the Health Protection Agency (HPA) monitored case estimates over the whole period of the pandemic, and published its findings online in its ‘Weekly Pandemic Flu Media Updates’ and its ‘Weekly National Influenza Reports’ [2], [6]. The last pandemic flu media update was published on 14 January 2010, the official end of the pandemic in England [2], [6]. By this time HPA's estimates totalled 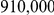 cases over the course of the pandemic in England [2]. This amounts to less than 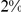 of the English population [7], a much lower figure than expected even with a basic reproductive number from the lower end of the published estimates.

This large discrepancy between the predicted and observed epidemics could have several causes. The basic reproductive number might have been very much smaller than estimated, a large part of the population might have been immune before the pandemic arrived, or many susceptible individuals might have been infected and become immune without being ill enough to register in the case estimates.

In what follows we use simple mathematical models to reproduce the HPA case estimates and try to learn how each of these three factors contributed to the surprisingly small epidemic. We then ask at what point in time during 2009 it should have become apparent that the English epidemic would be so small.

## Methods

### Phases of the Epidemic

We use the HPA case estimates which were made available to the public via the internet [2] as a data source for estimated numbers of symptomatic cases with influenza A(H1N1) in England. On 27 April 2009, the HPA announced the first laboratory confirmed infected case in the UK. It updated the number of new laboratory confirmed cases in England on a daily basis until 2 July 2009 when it switched to weekly updates of estimated numbers of new cases in the previous week. The last pandemic media update was published on 14 January 2010, when the influenza epidemic in England was officially declared to be over [6].

HPA's case estimates were an estimate of the number of people with symptomatic infections. The number of cases was estimated as the product of four factors: the number of GP visits with influenza-like-illness, the proportion of diagnosed cases that were confirmed as infected with A(H1N1), the proportion of symptomatic cases likely to contact the health services and the likely impact of the National Pandemic Flu Service when it became available [8], [9].

**Figure 1:**
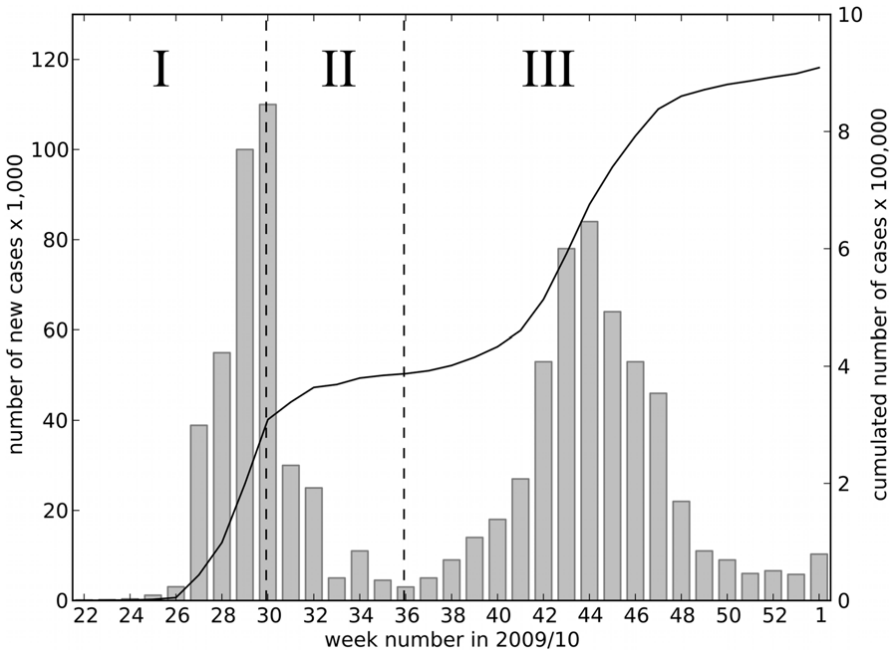
HPA case data and cumulative number of cases. The bars show the number of estimated new cases per week. The cumulative number of cases per week is presented as black line on a separate axis. The plot is separated into three phases, I–III, divided by dashed lines in week 30 and 36 between which state schools in England were closed [10]. Phase I is the initial growth phase before 22 July 2009, phase II is the school holiday phase until 3 September 2009, and at this date phase III with another growth and decline begins.

The data fall into three distinct phases:

**Phase I:** The first phase is the initial growth phase. It starts in week 19 with the first infected cases in the UK, and ends in week 30. From week 19 to week 27, the HPA released case reports based on laboratory confirmed cases. It changed its method to estimate cases using surveillance measurements in week 28 [2], explaining the jump in new cases between week 27 and 28. However, we expect that the laboratory confirmed case numbers released before week 28 are under-estimates of actual case numbers, because of limited laboratory facilities and the time delay to test for influenza A(H1N1).
**Phase II:** From week 30 to week 36 the number of cases declined. This is the period of school summer holidays in England. State schools were closed from 22 July to 3 September 2009 [10], exactly corresponding to phase II and marking the borders of the three phases. It is very likely that school closures during the summer break caused the decline in transmission events as children and young adults have been identified as the main age groups that were infected (see Figure 1 and [11], [12]). Most universities were also shut during this phase, reducing mixing amongst young adults.
**Phase III:** The last phase is the second epidemic wave after the school holiday. Schools reopened in week 36, and the number of new infections increased until week 44 after which numbers of new cases decreased again. The third phase ends in week 1 of 2010 with no more published HPA updates on estimated A(H1N1) cases.

### Theoretical Model

We developed a simple version of the SIR mathematical model that allows for two routes to immunity; one with disease and one without. As we are interested in short-term epidemic behaviour it is a model for a closed population without any births. There are four types of individual: susceptibles, 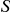, catch the infection at a rate that is proportional to the fraction of the population that is infectious. Upon infection they become infectious, either with symptoms, 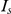, or without, 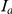. The proportion that develop symptoms on infection is 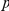. After a period of infectiousness they recover to their respective immune class, 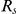 or 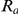. The rate of transmission of infection from those infected to the susceptibles is reduced by some fixed factor 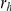 during phase II, the school holidays. At the start of the epidemic in England most people are susceptible so in class 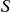, but some may already be immune so are already in class 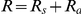. This model has five parameters: the proportion initially immune, 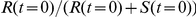, the transmission rate, 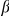, the reduction in transmission during the holidays, 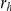, the duration of infectiousness, 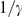, and the proportion of the population who become symptomatic if infected, 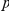:
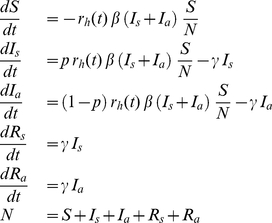
(1)where the reduction in transmission is a time dependent function:
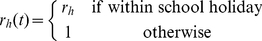
(2)Furthermore, the basic reproductive number can be defined as 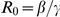, and the effective reproductive number is just 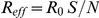. In the special case 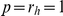 our model reduces to the standard, textbook epidemiological model called the “SIR model” [4].

### Serological Data

We used serological data based on samples drawn in England between July and September 2009. These were published in 2010 [11]. Table S1 shows the reported serological data and the demographic data that we used to calculate numbers immune before and after the first wave of the pandemic. The original report of the serological data split England into two areas, one with a high risk of seroconversion and one with a low risk. For our purposes we combine these using population data of the English population from the Office of National Statistics [7]. This yields estimates of the proportion of the population immune before the pandemic arrived and the proportion immune at the end of the first wave of the pandemic. The simple model that we use to understand these data is age-independent, and we therefore need to calculate proportions of the total population immune without age-stratification. To do that from age-stratified data we used demographic data on the current age and regional distribution [7].

### Qualitative Analysis

Our Method proceeds in four steps:

First we calculate how many people were immune to A(H1N1) before the pandemic and in September 2009. The difference between these two numbers yields the total number of people who were infected during the English epidemic up to the time of sampling. This is a conservative estimate as people might have been infected between their sampling, which started in mid-July, and September 2009. Our method therefore underestimates the actual number of infected cases.
Second we compare the number of seroconverters with the cumulative number of estimated symptomatic cases by the beginning of September to get an estimate of the proportion of all infections that manifest themselves in disease (parameter 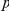 in the mathematical model). This parameter is calculated as a central value with upper and lower confidence bounds.

We then use these two pieces of information (proportion immune before the pandemic arrived and proportion of all infections manifesting as reported cases) and fit our model to the case report data. In fitting the model to the data we infer values for the three free model parameters: the basic reproductive number (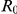), the infectious period (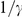) and the reduction in transmission during the summer holidays (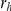).

In a fourth step we use our model to ask if it could have been obvious from case-reports alone that the English epidemic would be so small, and if so, when. If we assume 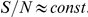, the number of symptomatic infected can be expressed through an exponential growth:
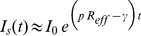
(3)The HPA data on symptomatic cases is based on weekly updates. This weekly sampling rate might be too broad to estimate the correct number of symptomatic cases at any time point, especially if the generation time is smaller than a week. But it allows us to evaluate the accumulated sum of symptomatic cases (new infections plus recovered ones), which grows with:
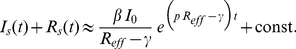
(4)where the last term expresses the normalisation constant. As before, the growth is described with an exponential function. We can determine the effect of the depletion of susceptible hosts, or in other words the difference in 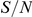 between the two waves, by taking the natural logarithm of the accumulated sum of HPA's estimates. The slope of the logarithmic growth periods is (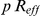 - 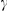), so differences in the slope of the two epidemic waves reflect the drop in 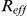 caused by a depletion of susceptible hosts from one wave to the next.

## Results

The English epidemic unfolded in three phases (Figure 1). The first phase of rapid growth was followed by a second phase when the number of cases fell as schools closed for the summer holiday. The third phase saw the start of the second wave as schools reopened in the autumn. After a short period of exponential growth, the second wave peaked in mid-November and then tailed off. This was surprisingly early, given the predicted attack rates.

These data raise four questions:

1. How transmissible was this strain of influenza amongst the population of England (i.e. how big was 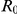)?
2. By how much did transmission decline during the summer holidays (i.e. what was 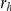)?
3. For each case detected how many seroconverters were there (i.e. what was 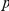)?
4. How soon could the failure to develop into a large epidemic have been recognised?

**Figure 2:**
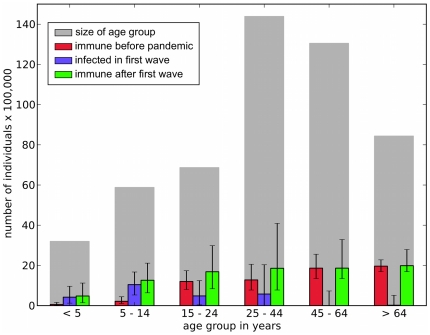
Comparison of serological data by age group. The number of individuals in each age group is shown with large, grey bars. Red bars show the number of individuals immune before the pandemic. Blue bars represent numbers who seroconverted between April and September 2009. Green bars show the number immune by early September 2009. All numbers are calculated using serological data and demographic data from England (see Table S1).

These questions can be addressed by combining the case report data with serological data (Figure 2). Those serological data show (red bars) that a substantial number of people were immune before the epidemic arrived and that this was particularly true for those over the age of 45. Correspondingly there were many seroconversions by early September (blue bars) in the young and very few in those over 45. Finally by early September a large part of the English population was already immune (green bars), regardless of age.

Adding up the number of serconverters across ages gives an estimate of 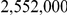 infections in England by the beginning of September 2009 (range 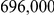
–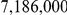 based on 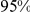 confidence intervals for the published serological studies). Yet, by that time, only 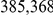 symptomatic cases had occurred according to HPA case estimates. We can take the ratio of symptomatic cases to all infections as an estimate of the proportion of cases that are symptomatic, parameter 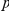 in our model. The serological data imply that 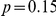 (range 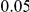
–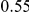). This is more easily interpreted as the inverse of 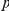; the number for seroconverters for each recorded case. This has a mean of 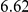 with range 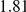
–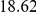. In what follows we use four different values of 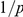: 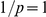, every seroconverter is symptomatic; 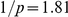, the lower bound estimate from the serological data; 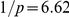, the mean from the serological data; and 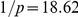, the upper bound from the serological data.

The serological data also yield an estimate for the proportion of the population who were immune to the pandemic influenza strain before it arrived. The estimate is 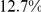 and is used to define initial conditions for the model in equation (1).

**Figure 3:**
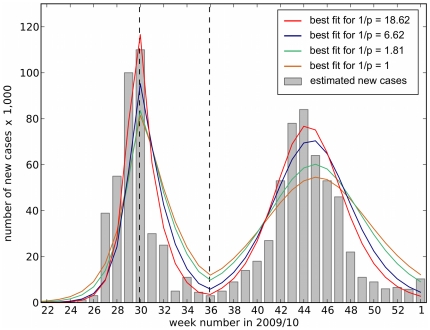
Comparison of HPA case estimates with theoretical predictions of new cases. The HPA case estimates per week are represented in a bar plot. The model in equation (1) was fitted to these data with state variable 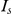 representing reported cases. The initial conditions were set at the observed value of 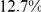 of the population immune before week 22. Four different values of parameter 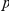 were assumed corresponding to four different fits: 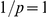 - brown line, 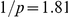 - green line, 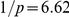 - blue line and 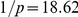 - red line. The figure illustrates that 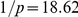 gives the best agreement between model and data with the smallest least square error, and allows our model to reproduce the case estimates. The fitting procedure yields estimates for the model's three parameters, shown in Table 2. Only when 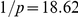 do these parameter estimates agree with previously published values, compare Table 2 with Tables 1, 3 and 4.

Having made estimates for the initial conditions and the ratio of seroconverters to cases we proceed to fit the model to the case data. This amounts to finding a set of parameter values that are consistent both with the case reports and with the serological data. Figure 3 presents the best fits of our mathematical model to the case reports for four different values of 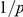 ranging from the situation where all seroconverters present as cases (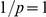) to the upper limit of estimated values in which for each reported case there are more than 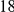 seroconverters (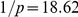) [11]. All fits assume that 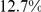 of the population are immune before the pandemic arrives, reflecting pre-existing immunity in the population. Our fitting procedure is to fix 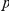 to one of our four values, fix the percentage immune before the pandemic to 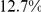 and then estimate the three parameters 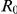, 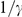 and 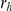 by making a least square fit of the solution of the model (equations (1) and (2)) to the HPA's case report data. Table 2 reports best fit parameter estimates for each value of 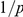. Overall, the best fit is for 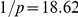. The parametric fit yields 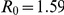, very much in agreement with previous estimates (see Table 1). In addition, an infectious period of 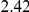 days lies well within previous independent estimates for influenza A(H1N1) (see Table 3 and Table 4). The best-estimate reduction factor, 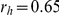 reveals that transmission is 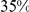 lower over the period of school holidays. While there is no empirical data to validate this result, it seems plausible as the number of infected cases was highest in the age group of 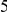 to 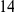 years old (see Figure 2). This reconciliation of the case report data and the serological data concludes that the 2009 English epidemic of symptomatic cases was small for the following reasons:

1. a large fraction (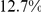) of the population were immune before the pandemic arrived in England,
2. for every reported case there were many (approx. 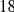) seroconversions that were not reported as cases so the susceptible pool was very quickly diminished,
3. initial estimates for 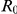 of around 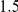 were roughly right.

**Table 2:** Estimated parameters using a least square fit.

Estimated parameters			
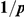	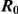	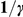	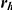
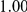	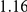	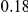	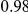
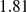	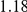	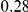	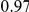
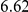	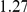	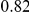	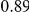
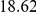	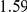	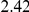	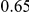

All parameters were estimated assuming an immune fraction of 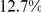 at the beginning of the pandemic. A least square fit was used to minimise the error between our model and HPA case estimates.

**Table 3:** Infectious period estimates for influenza A(H1N1) from two independent studies with confidence interval (CI).

Infectious period
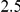 (CI: 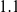 – 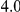 ) [19]
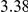 (CI: 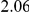 – 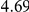 ) [24]

The infectious period is presented in days.

**Table 4:** Generation time estimates for influenza A(H1N1) from several independent studies.

Generation time
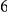 – 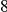 [25]
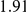 (CI: 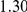 – 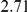 ) [1]
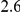 (CI: 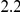 – 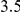 ) [26]
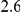 – 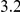 [17]
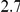 (CI: 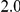 – 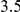 ) [27]
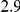 [21]
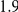 – 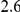 (CI: 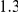 – 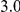 ) [22]
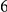 (CI: 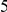 – 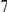 ) [23]

The generation time is presented in days. A range of possible generation times is shown when multiple estimations were made. The confidence interval (CI) is given wherever possible. A median latent period of 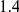 days (CI: 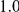
–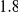) [27] and 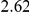 days (CI: 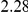
–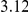) [24] has been reported. The infectious period can be estimated using the generation time as latent period and half the infectious period are on average the generation time [4].

**Figure 4:**
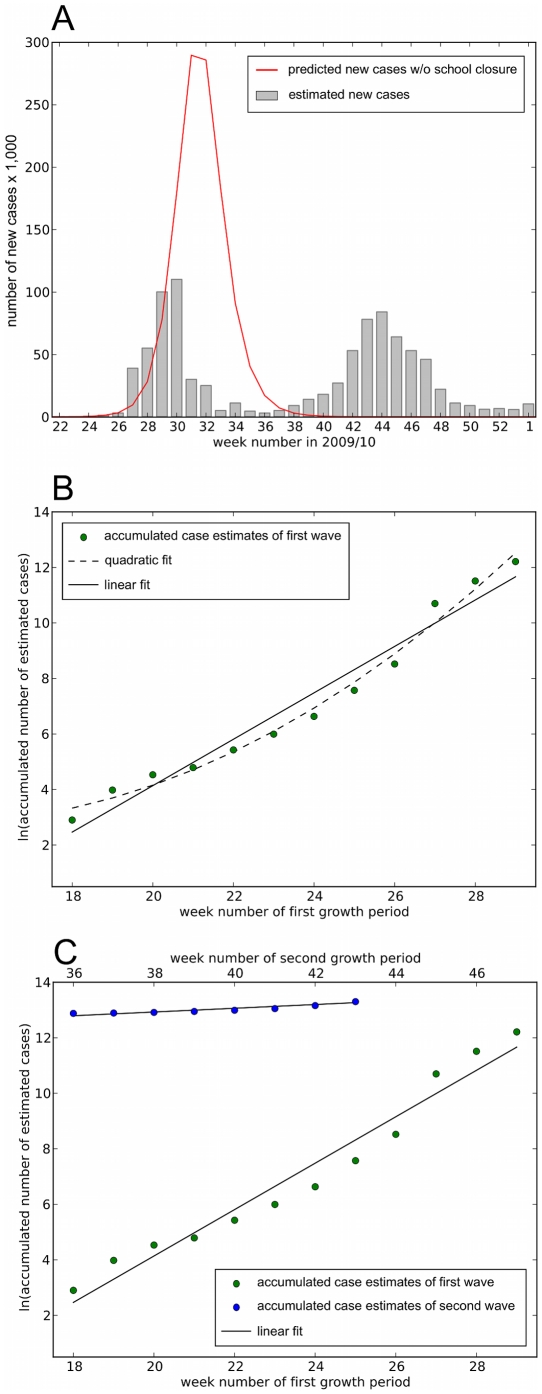
When did growth data reveal that the English epidemic would be small? A. shows that it was unlikely that the first wave would indicate that susceptible hosts were becoming exhausted. If there had been no school break we expect that the epidemic would have gone on growing for several weeks after week 30 when they actually shut. The red line shows predicted dynamics of the English epidemic if the schools had not closed (i.e. if 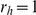 and other parameters are the best fits for the model of equation (1)). B. This is confirmed in regression analyses of the natural logarithm of the cumulative number of cases. Data are filled circles, linear fit is the solid line and quadratic fit is the dashed line. Linear fit: ln(cumulative cases) 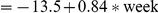. Quadratic fit: ln(cumulative cases) 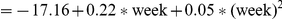. The quadratic term is significant (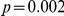) but small and positive, signifying that, if anything, the epidemic was accelerating just before the schools broke up for summer. C. compares growth rates of the first wave (green dots) and second wave (blue dots). An analysis of covariance reveals that the two slopes are significantly different (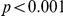). Linear fit for second wave: ln(cumulative cases) 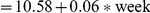.

This raises the following question: how early would it have been possible to detect that there were so many seroconverters for every reported case? Was the epidemic already starting to slow in late July, before schools broke up? Figure 4 A answers this question with a clear “no”. If the schools had not broken up, using our best-fit parameters, we believe that the epidemic would have continued to grow rapidly for several more weeks. It is therefore not surprising that a logarithmic plot of cumulated cases during the weeks before schools closed (Figure 4 B) shows that the epidemic was clearly still in its exponential growth phase with no indication of a falling off in the growth rate. However, when schools reconvened in September, the second pandemic wave clearly grew at a much slower rate than before the holiday. Figure 4 C plots cumulated case numbers in a logarithmic plot that compares the rising phases of the first and second waves. The second wave is clearly growing very much more slowly than the first wave, which is consistent with the large fraction of the population that had already seroconverted by the end of the first wave. In short, the very slow growth of the second wave of the pandemic was a clear indicator that many people had already seroconverted during the first wave.

## Discussion

The HPA published their case estimates on influenza A(H1N1) infections over the period of the pandemic in England. Overall, the HPA estimated that approximately 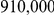 individuals were ill with the pandemic influenza virus during 2009, amounting to less than 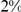 of the English population [2]. This surprisingly small number of symptomatic cases only makes sense in the light of the HPA's serological survey which found that around 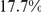 of the English population were already immune by the end of the first wave of the pandemic in late September 2009. This observation, combined with knowledge of the level of immunity prior to the pandemics' arrival and the dates of the English summer holidays combine together to tell a coherent story about the 2009 influenza pandemic in England. The pandemic had a comparatively small impact because for every case that was reported there were around 18 seroconversions with no symptoms at all.

In retrospect, it was clear from the very slow growth of the second epidemic wave that something was different as the known cases up until September were too few to explains such slow growth. Depletion of susceptibles through asymptomatic cases would always have been a strong contender as a mechanism for this slow observed growth.

The HPA's serology study was organised, collected, analysed and published in a very short space of time. No other country has, to our knowledge, published such extensive data [13]–[15]. In principle, the same pattern that was picked up in these data in September would have been seen in samples drawn even earlier. This modelling study is a ringing endorsement of the explanatory power of serological surveys in a pandemic setting. However, it is not obvious that a serological survey could have been organised and executed any earlier in a pandemic than this one was.

The distinction between infection and disease is an early lesson in the teaching of infectious disease biology [16]. Predictions of the likely impact of the 2009 influenza pandemic were made using models that did not distinguish between cases of infection and cases of disease. A very simple model that allows asymptomatic seroconversion is presented here and yields a coherent reconciliation of the estimated numbers of disease versus the estimated number of infections through the course of the 2009 pandemic in England. Once it is clear how many asymptomatic cases there were, the epidemiology of the 2009 influenza pandemic in England makes sense.

## Supporting Information

Table S1Shown are the serological data of [11] combined with the demographic data of England given by [7]. Values in brackets show the 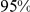 confidence intervals as estimated by [11]. All numbers shown are in 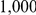
s. Numbers might not sum due to rounding.(PDF)

## References

[1] Fraser C, Donnelly CA, Cauchemez S, Hanage WP, Van Kerkhove MD (2009). Pandemic potential of a strain of inuenza A (H1N1): early findings.. Science.

[2] Health Protection Agency (2009). Weekly National Inuenza Reports.. http://www.hpa.org.uk/web/HPAweb&HPAwebStandard/HPAweb_C/1244442494458.

[3] Donaldson LJ, Rutter PD, Ellis BM, Greaves FEC, Mytton OT (2009). Mortality from pandemic A/H1N1 2009 inuenza in England: public health surveillance study.. BMJ.

[4] Anderson RM, May RM (1992). Infectious Diseases of Humans: Dynamics and Control (Oxford Science Publications).

[5] Scherer A, McLean AR (2002). Mathematical models of vaccination.. Br Med Bull.

[6] Health Protection Agency (2009). Weekly Pandemic Flu Media Update.. http://www.hpa.org.uk/NewsCentre/NationalPressReleases/2009PressReleases/.

[7] Office for National Statistics (2009). 2009 Population Estimates in England.. http://www.statistics.gov.uk/popest.

[8] Health Protection Agency (2009). Synopsis of the method used to estimate the number of pandemic inuenza (H1N1) 2009 cases in England in the week 21 to 27July 2009.. http://www.hpa.org.uk/web/HPAweb&HPAwebStandard/HPAweb_C/1244442494458.

[9] Health Protection Agency (2009). Method used to estimate new pandemic (H1N1) 2009 inuenza cases in England in the week 3 August to 9 August 2009.. http://www.hpa.org.uk/web/HPAweb&HPAwebStandard/HPAweb_C/1244442494458.

[10] Directgov (2009). School Term Dates.. http://www.direct.gov.uk/en/Parents/Schoolslearninganddevelopment/SchoolLife/DG_4016103.

[11] Miller E, Hoschler K, Hardelid P, Stanford E, Andrews N (2010). Incidence of 2009 pandemic inuenza A H1N1 infection in England: a cross-sectional serological study.. Lancet.

[12] Girard MP, Tam JS, Assossou OM, Kieny MP (2010). The 2009 A (H1N1) inuenza virus pandemic: A review.. Vaccine.

[13] Chen H (2009). Serologic Survey of Pandemic (H1N1) 2009 Virus, Guangxi Province, China.. Emerg Infect Dis.

[14] Prachayangprecha S, Makkoch J, Payungporn S, Chieochansin T, Vuthitanachot C (2010). Serological analysis of human pandemic inuenza (H1N1) in Thailand.. J Health Popul Nutr.

[15] Delangue J, Salez N, Ninove L, Kieffer a, Zandotti C (2011). Serological study of the 2009 pandemic due to inuenza A H1N1 in the metropolitan French population.. Clin Microbiol Infect.

[16] Mims CA, Nash A, Stephen J (2000). Mims' Pathogenesis of Infectious Disease.

[17] Yang Y, Sugimoto JD, Halloran ME, Basta NE, Chao DL (2009). The transmissibility and control of pandemic inuenza A (H1N1) virus.. Science.

[18] Nishiura H, Chowell G, Safan M, Castillo-Chavez C (2010). Pros and cons of estimating the reproduction number from early epidemic growth rate of inuenza A (H1N1) 2009.. Theor Biol Med Model.

[19] Balcan D, Hu H, Goncalves B, Bajardi P, Poletto C (2009). Seasonal transmission potential and activity peaks of the new inuenza A(H1N1): a Monte Carlo likelihood analysis based on human mobility.. BMC Med.

[20] Munayco CV, Gómez J, Arrasco J, Kochel TJ, Fiestas V (2009). Epidemiological and trans- missibility analysis of inuenza A(H1N1)v in a southern hemisphere setting: Peru.. Euro Surveill.

[21] McBryde E, Bergeri I, Gemert CV, Rotty J, Headley EJ (2009). Early transmission characteristics of inuenza A(H1N1)v in Australia: Victorian state, 16 May–3 June 2009.. Euro Surveill.

[22] de Silva UC, Warachit J, Waicharoen S, Chittaganpitch M (2009). A preliminary analysis of the epidemiology of inuenza A(H1N1)v virus infection in Thailand from early outbreak data, June–July 2009.. Euro Surveill.

[23] Pourbohloul B, Ahued A, Davoudi B, Meza R, Meyers LA (2009). Initial human transmission dynamics of the pandemic (H1N1) 2009 virus in North America.. Inuenza Other Respi Viruses.

[24] Tuite AR, Greer AL, Whelan M, Winter AL, Lee B (2010). Estimated epidemiologic parameters and morbidity associated with pandemic H1N1 inuenza.. Can Med Assoc J.

[25] Dawood FS, Jain S, Finelli L, Shaw MW, Lindstrom S (2009). Emergence of a novel swineorigin inuenza A (H1N1) virus in humans.. N Engl J Med.

[26] Cauchemez S, Donnelly CA, Reed C, Ghani AC, Fraser C (2009). Household transmission of 2009 pandemic inuenza A (H1N1) virus in the United States.. N Engl J Med.

[27] Lessler J, Reich NG, Cummings DaT, Nair HP, Jordan HT (2009). Outbreak of 2009 pandemic inuenza A (H1N1) at a New York City school.. N Engl J Med.

