# Supplementary material for: Why Was the 2009 Influenza Pandemic in England So Small?
Source: PLoS One. 2012 Feb 10;7(2):e30223. doi: 10.1371/journal.pone.0030223 (PMC3277589; doi:10.1371/journal.pone.0030223)
Supplement: Table S1 — Shown are the serological data of [11] combined with the demographic data of England given by [7]. Values in brackets show the confidence intervals as estimated by [11]. All numbers shown are in s. Numbers might not sum due to rounding. (PDF) [file pone.0030223.s001.pdf]

Combining serological and demographic data									
Age group	English population		% immune before pandemic	% immune after first wave		Difference September 2010 - baseline		Number immune September 2010	Number infected after first wave
	High risk group	Low risk group		High risk group	Low risk group	High risk group	Low risk group		
< 5	1,222.5	1,973.5	1.8 (0.6 - 5.0)	23.1 (11.0 - 42.1)	10.0 (3.5 - 25.6)	21.3 (8.8 - 40.3)	8.2 (0.9 - 23.9)	57.5 (19.2 - 159.8)	422.2 (125.3 - 964.3)
5 – 14	2,020.1	3,859.6	3.7 (1.8 - 7.5	45.7 (30.5 - 61.8)	8.8 (4.5 - 16.4)	42.0 (26.3 - 58.2)	5.1 (-0.6 - 12.9)	217.5 (105.8 - 441.0)	1,045.3 (531.3 - 1,673.6)
15 – 24	2,353.3	4,512.8	17.5 (11.7 - 25.3)	38.1 (20.8 - 59.1)	12.9 (7.5 - 21.2)	20.6 (1.6 - 42.4)	-4.6 (-14.0 - 5.5)	1,201.6 (803.3 - 1,737.1)	484.8 (37.7 - 1,246.0)
25 – 44	5,657.2	8,728.4	8.9 (5.4 - 14.3)	15.1 (7.9 - 27.1)	11.5 (6.5 - 19.4)	6.2 (-2.8 - 18.7)	2.6 (-4.8 - 11.2)	1,280.3 (776.8 - 2,057.1)	577.7 (0.0 - 2,035.5)
45 – 64	4,123.4	8,924.5	14.3 (10.3 - 19.6)	11.5 (6.2 - 20.5)	11.1 (6.6 - 18.1)	-2.7 (-10.3 - 7.1)	-3.2 (-10.1 - 4.9)	1,865.8 (1,343.9 - 2,557.4)	0.0 (0.0 - 730.1)
65 >	2,484.9	5,949.6	23.3 (20.0 - 27.0)	24.2 (15.2 - 36.2)	18.9 (13.6 - 25.6)	0.9 (-8.8 - 13.3)	-4.4 (-10.8 - 3.1)	1,965.2 (1.686.9 - 2,277.3)	22.4 (0.0 - 514.9)
Total	17,861.5	33,948.3	-	-	-	-	-	6,588.1 (4,736.0 - 9,229.7)	2,552.3 (696.1 - 7,186.4)
